# Transcriptional Regulation of Abscission Zones

**DOI:** 10.3390/plants8060154

**Published:** 2019-06-06

**Authors:** Joonyup Kim, Jong-Pil Chun, Mark L. Tucker

**Affiliations:** 1 Department of Horticultural Science, Chungnam National University, 99 Daehak-ro, Yuseong-gu, Daejeon 34134, Korea; jpchun@cnu.ac.kr; 2 Soybean Genomics and Improvement Laboratory, Agricultural Research Service, USDA Bldg. 006, 10300 Baltimore Ave., Beltsville, MD 20705, USA; MarkLeoTucker@gmail.com

**Keywords:** abscission, abscission zone, transcriptional regulation, regulatory modules, comparative analysis, extracellular matrix, boundary layer

## Abstract

Precise and timely regulation of organ separation from the parent plant (abscission) is consequential to improvement of crop productivity as it influences both the timing of harvest and fruit quality. Abscission is tightly associated with plant fitness as unwanted organs (petals, sepals, filaments) are shed after fertilization while seeds, fruits, and leaves are cast off as means of reproductive success or in response to abiotic/biotic stresses. Floral organ abscission in Arabidopsis has been a useful model to elucidate the molecular mechanisms that underlie the separation processes, and multiple abscission signals associated with the activation and downstream pathways have been uncovered. Concomitantly, large-scale analyses of omics studies in diverse abscission systems of various plants have added valuable insights into the abscission process. The results suggest that there are common molecular events linked to the biosynthesis of a new extracellular matrix as well as cell wall disassembly. Comparative analysis between Arabidopsis and soybean abscission systems has revealed shared and yet disparate regulatory modules that affect the separation processes. In this review, we discuss our current understanding of the transcriptional regulation of abscission in several different plants that has improved on the previously proposed four-phased model of organ separation.

## 1. Introduction

The plant architecture is continuously being shaped and reshaped by assembly and modification of cell wall materials that consist mainly of celluloses, hemicelluloses (cross-linking glycans), pectins, lignin, and structural proteins. Regulated restructuring of these components within the wall matrix is a basis for plant development and its response to environmental challenges such as plant-pathogen interactions. Organ separation (abscission) is a part of the dynamic nature of plant architecture and reproductive development that involves changes in cell function and cell wall structure. The cellular processes that ultimately lead to separation take place in a developmentally defined region of cells called the abscission zone (AZ, or the target cells) at the base of the organ to be shed [1]. Abscission of the plant organ (e.g., leaf, flower, fruit, petal, etc.) occurs when the organ is no longer beneficial to the survival of the parent plant or as a step in reproductive development [2,3,4,5,6,7].

**Figure 1:**
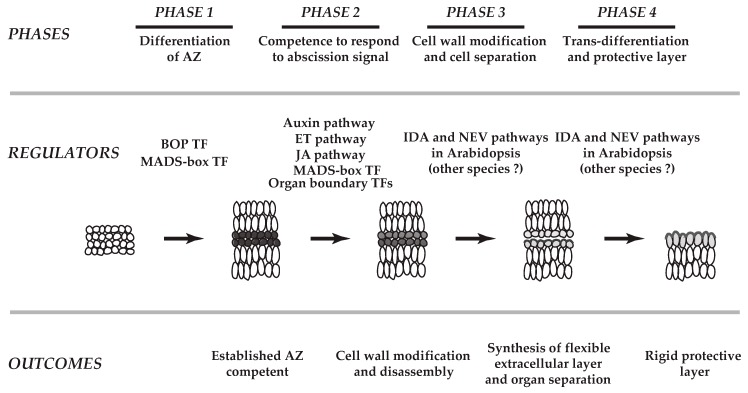
A schematic model of abscission in plants. The working model for abscission consists of largely four basic phases. First, differentiation of abscission zone (AZ); second, acquisition of the competence of the AZ to respond to abscission signals (e.g., decline in auxin); third, cell wall modifications and cell separation; and fourth, trans-differentiation of the AZ and formation of a protective layer. Based on recent transcriptome analyses (Kim et al., 2015, Kim et al., 2016), roles of transcription factors (TFs) that define the boundary layer cells in the AZs (Organ boundary TFs, Phase 2) and genes linked to the synthesis of flexible extracellular matrix (outcome of Phase 3) are implemented on the previously proposed four phases of separation processes (modified from Patterson, 2001 and Kim, 2014). In Phase 1, both tomato (Xu et al., 2016) and Arabidopsis (McKim et al., 2008) BOP TFs, and a tomato MADS-box TF of JOINTLESS (Mao et al., 2000) are known to be critical in establishment of AZ. MADS-box TFs (e.g., AGL15, AGL18, AGL24) affect timing of abscission in Arabidopsis (Phase 2). In addition, a membrane traffic regulator (NEVERSHED, NEV) and a small signaling peptide (INFLORESCENCE DEFICIENT IN ABSCISSION, IDA) are associated with cell wall disassembly and modifications in Phase 3 and Phase 4 of Arabidopsis, but their specific roles in other species have not been determined.

Abscission has been studied for more than 170 years [8]. It has been nearly 100 years and more than 50 years since the plant hormones ethylene and auxin, respectively, were found to play a role in the abscission process [1,9]. To date, nearly all of the classical plant hormones (i.e., cytokinins, gibberellins, jasmonic acid, abscisic acid, and brassinosteroids) have been demonstrated to affect the timing of abscission [1,4,7,10,11,12,13,14]. In recent years, many additional signaling components including a small signaling peptide, receptor-like kinases, MAP kinases, transcription factors, and membrane traffic regulators have been shown to be critical to differing phases of separation processes [3,5,6,15,16,17,18,19,20,21,22,23,24,25,26,27,28,29,30,31,32,33,34]. The working model for abscission consists of largely four basic phases [2,4,5,7,10,12,23,30,35,36,37]: first, differentiation of abscission zone (AZ); second, acquisition of the competence of the AZ to respond to abscission signals (e.g., decline in auxin); third, cell wall modification and cell separation; and fourth, trans-differentiation of the AZ and formation of a protective layer (Figure 1).

Over the past few decades, advances in genetics and molecular and biochemical approaches have greatly enhanced our understanding of abscission. The advent of Arabidopsis as a model plant has led to significant progress in understanding of the regulatory mechanisms evoked in the abscission of floral organs (petals, sepals and stamens) [7,12,23]. Recent technological breakthroughs in large-scale studies of transcriptomes, proteomes, and metabolomes have accelerated the identification of molecular pathways utilized in various abscission systems (e.g., leaf, flower, fruit) of different plant species [6,38,39,40,41,42,43,44,45]. These studies revealed that there exist common signals, including ethylene and auxin, that evoke both conserved regulatory pathways and divergent co-regulators, which modulate organ separation (e.g., leaf, flower, fruit) across plant species (e.g., Arabidopsis, tomato (*Solanum lycopersicum*), soybean (*Glycine max*)). Herein, we discuss our current understanding of transcriptional regulation in abscission in a diverse set of plants, and how recent insights into these regulatory mechanisms have improved on the previously proposed four-phased model of organ separation (Figure 1) [2,7,12].

## 2. Cellular Changes in Various Abscission Systems

Anatomical and cytological features in the AZ of many plant species are well documented [1]. In order for a plant to cast off an organ (e.g., leaves, floral parts, whole flowers, or fruit) in response to developmental changes or environmental challenges (i.e., heat, drought, frost), at least two major biochemical properties within the AZ are required to be altered. The first and most obvious change is the breakdown of the wall matrix that provides structure to the cells and tissues within the AZ. The middle lamella, the biological glue that connects cells to each other, is primarily composed of pectins [46]. Breakdown of the middle lamella is associated with expression of a variety of pecitnases, both polygalacturonases (PGs) and pectate lyases (PLs), and also pectin methylesterases (PMEs) that are proposed to open up a pectin matrix by reducing esterification and cross-linking of pectin polymers for access by other degradative enzymes, i.e., PGs and PLs [11,47,48,49,50,51,52]. In addition to breakdown of the middle lamella, the primary cell wall surrounding each cell must also be modified to loosen the rigid cell wall structure to allow cells to expand, which creates the forces required to push apart proximal and distal tissues between the parent plant and the organ being shed [1,10,11]. The primary cell wall consists of celluloses, hemicelluloses (cross-linking glycans), pectins, and structural proteins [46]. Chemical properties of the AZs are modified by cell wall modifying proteins including pecitnases (PGs, PLs), cellulases (beta-1,4-endogulcases, CELs), expansins (EXPs), and xyloglucan endotransglucosylase/hyrolases (XTHs) [10]. In addition to changes in pectin metabolism facilitated by PGs, PLs, and pectin methylesterases (PMEs) [11,47,48,49,50,51,52] which are also essential to middle lamella degradation, it has been demonstrated that the hydrolysis of celluloses (or cellulosic microfibrils) is also needed for a successful separation process [11,53]. The hydrolysis of celluloses appears to be required for cell expansion coordinated by other enzymes like EXPs and XTHs [11,54]. The loosening of the cell wall is accompanied by an increase in cell turgor resulting from hydrolysis of starch in the AZ cells [55]. Although the middle lamella and primary cell walls of higher plants are all composed of related chemical polymers, the actual composition and structure can vary considerably from one plant or AZ to another [56]. Thus, it might be predicted that the expression of genes associated with modification and degradation of the wall matrix would be different in the various AZs.

Secondly, but equally important to a successful organ separation process, is the formation of a protective layer to limit water loss and cast a physical barrier against opportunistic pathogen attacks [1]. Synthesis of the protective layer commences as cell separation unfolds in the AZ and continues after organ separation is complete. In support of anatomical observations for the formation of the protective layer in the AZ [1], it has been shown that within the AZ there is an increase in the activity of stress-related peroxidases which have been suggested to play a role in the lignification of the AZ, IAA oxidation, and gene expression associated with stress responses [11,57,58]. The formation of the protective layer in the AZ of herbaceous and woody plants is more pronounced on the proximal side of the AZ than the distal side [11,57,58]. Nonetheless, it was observed that early lignification of the Arabidopsis floral organ AZ was most prominent on the distal side of the AZ, which, rather than protecting cells from pathogen attack, may be playing a role in restricting cell expansion in the distal cells to create the differential forces required to push away the distal organ for separation [59].

From a strictly applied perspective of improving agricultural economics by improved control of abscission, the best condition to study cellular changes in abscission might be under field conditions. However, controlled environments made possible in a greenhouse and a growth chamber to control environmental parameters, e.g., temperature, light, and water are of great benefit to produce reproducible experimental results. In addition, explant systems are often used wherein a portion of the plant that includes the AZ, e.g., inflorescence, is excised from the parent plant and then exogenously treated with hormones and/or chemicals that alter specific biochemical processes in the AZ. These in vitro explant systems are commonly used for work with tomato, soybean, bean, and coleus [1,11,57]. One of the most common requirements for the initiation of abscission is a decline of auxin in the AZ. In these explant systems this can be done by removing the distal organ, which is a large source of auxin that moves basipetally towards the AZ and inhibits abscission. For example, it is common practice to remove the flower from the tomato pedicel leaving approximately 2 mm of pedicel distal to the AZ, or removal of the leaf blade distal to the leaf petiole, which contains the AZ [12,57]. Therefore, as abscission is a developmentally programmed process that is readily influenced by environmental changes, studies with intact plant systems (Arabidopsis floral organs) in controlled environments and the explant systems (tomato flower pedicel explants) are used to uncover the molecular mechanisms associated with abscission because they provide the platform that generates reproducible and statistically sound data.

## 3. Comparative Analysis of Transcriptomes in Diverse Abscission Systems

### 3.1. Variability in Cell Wall Disassembly

Abscission behaviors differ in many plant species [1] and, as expected, the changes in gene expression associated with the separation processes are complex and varied in different abscission systems [43]. There may be several reasons for this diversity. Although it has been demonstrated that auxin plays an inhibitory role in many abscission systems, the requirement of ethylene in different abscission systems appears not to be the same. In soybean leaf and tomato flower abscission, it has been shown that ethylene is essential [6,60,61], while in Arabidopsis floral organ abscission ethylene controls the timing of organ separation [28,62]. Further, the abscission data obtained from different abscission systems can vary owing to dissimilar collection methods for the AZs. For instance, in soybean explant system, the AZ samples can be collected after removal of auxin source (e.g., leaf blade, see above) followed by treatment of explants with exogenous ethylene (25 μL/L) that both synchronizes and expedites the abscission process, while the AZ samples of tomato flower and Arabidopsis floral organs can be collected from inflorescences that may resemble a more natural abscission process [43]. Nonetheless, a recent comparative study between the AZs of soybean leaves, tomato flowers, and Arabidopsis floral organs revealed all three had overlapping and non-overlapping patterns in the regulation of gene expression. On the whole, there was a marked increase in expression of genes linked to cell wall disassembly, but the magnitude of expression for different wall modifying genes in each abscission system was varied, which is likely attributable to differences in the experimental design and the inherent nature of the system. Among many cell wall disassembly genes expressed in soybean and tomato explant systems, a surprisingly small number of *cellulase* genes (e.g., *GmCel01*, *SlCel1*) and a few *PGs* that constituted approximately 75% of all the AZ-specific *cellulase* and *polygalacturonase* genes were expressed within these two families [43]. These few cell-wall modifying genes within the two gene families would appear to play a major role in abscission. Nevertheless, within the cellulase and PG families there may be considerable functional redundancy, which suggests that cellular and temporal specific regulation of the promoters for each of the genes is the driving force for the evolution of a large gene family with gene products of similar function [63,64]. It is noteworthy that in contrast to the tomato and Arabidopsis abscission systems, where *XTH* gene expression is markedly up-regulated in an AZ-specific manner, in soybean *XTH* gene expression was not AZ-specific [46,65]. XTH is important for remodeling of the cellulose-xyloglucan network that renders the cell wall extensible [46,65]. Because the expression pattern of *XTHs* was not AZ-specific in soybean abscission, this suggests that *XTHs* may have a function in cell wall modification associated with senescence, which in the soybean explant system occurs in the non-AZ tissue (petiole) [46,65]. Curiously, when the expression of genes associated with cell wall disassembly was compared between the AZs of Arabidopsis wild-type plant and the non-abscising mutant, *haesa*/*haesa*-*like* 2 [43,66], the magnitude for the gene expression linked to cell wall disassembly (e.g., *cellulases* and *PGs*) was not as great as that seen in the tomato and soybean explant systems. In summary, although the gene expression associated with cell wall disassembly correlated with the progression of cell separation in diverse abscission systems, their temporal regulation, magnitude of expression, and AZ-specificity during abscission varied. Taken together, comparative analysis reaffirms the complex nature of regulation in cell wall disassembly necessary for diverse abscission systems.

### 3.2. Reconstruction of a Flexible Extracellular Matrix during Separation

The structural proteins of a typical primary cell wall consist of extensins, arabinoglactan proteins, proline-rich proteins, glycine-rich proteins, and hydroxyproline-rich glycoproteins [46]. Abundance and composition of these structural proteins in the cell wall differ depending on cell type and plant species. The composition of structural proteins that comprises the protective layer in the AZs presumably varies in different abscission systems. The exact composition of the protective layer that creates a barrier to pathogen attacks and reduces water loss is undetermined. Expression for genes related to cellulose synthesis, and structural proteins extensins, arabinoglactan proteins, proline-rich proteins, glycine-rich proteins, and hydroxyproline-rich glycoproteins was either decreased in the soybean system or largely unaltered in the tomato and Arabidopsis systems. Based on the expression data, cellulose synthesis and synthesis of these primary cell wall proteins are not likely typified in the formation of a protective layer in the abscission systems [43].

It has been shown that the deposition of callose, a 1–3 linked beta glucan polymer, is one of the plant defense responses [67,68]. Callose is commonly a major constituent of cell wall reinforcement at sites where the tissue has been damaged. Callose was observed to have an anti-microbial influence at the infection site of the host cell wall [69]. As gene expression profiles for callose synthesis were largely unaltered in the AZs of soybean leaves, tomato flowers, and Arabidopsis floral organs, callose did not appear to be a major component in the new extracellular matrix that could protect the separation layer cells [43].

The role of gene expression for Pathogenesis-Related (PR) proteins during abscission has been suggested to protect vulnerable abscising cells against opportunistic pathogens [6,10,70,71,72]. However, transcriptome profiling from the AZs of soybean leaves, tomato flowers, and Arabidopsis floral organs revealed additional aspects as to their function [43]. Comparative analysis of transcriptomes in the AZs of these three systems indicate that, in addition to what is proposed for these genes in an enzymatic role for the defense in the AZ [73], these *PR* genes may be part of the proteinaceous wall components in the protective layer of AZs [12,43]. As expected, overall patterns of gene expression increased as abscission progressed. In particular, expression of genes that encode thaumatin, chitinase, and beta-1,3-glucanse in the soybean leaf explant system increased in an AZ-specific manner during and after organ separation. In tomato flower pedicel abscission, gene expression for chitinase and kuntiz trypsin inhibitor proteins increased notably prior to the actual cell separation process and, similarly, in Arabidopsis floral organ separation, gene expression for thaumatin, chitinase, and kunitz trypsin inhibitor proteins was AZ-specific and also preceded organ separation. Of interest is that gene expression of *PAR1* (*photoassimilate-responsive-1*) genes with unknown enzymatic function [74] was strongly up-regulated in an AZ-specific manner in all three abscission systems. The common molecular features of structural proteins and the above PR proteins are predicted to be secreted as they contain N-terminal signal peptide sequences, and some are small proteins (15 to 25 kDa) that might be part of an extensible extracellular boundary layer on the surface of separating cells.

There is mounting evidence that gene expression of proteins associated with the synthesis of a cuticle-like substance, e.g., lipid transfer proteins (LTPs), and genes that affect the phenylpropanoid pathway are tightly associated with organ adhesion and abscission [75,76,77]. These studies demonstrate that the activity of these genes is crucial in formation and secretion of cuticle-like components deposited into the extracellular matrix of abscising cells. Coordinated regulation of cell separation and synthesis of a cuticular-like matrix are crucial for plant development and in interactions with environmental stresses [76,77]. Gene expression linked to synthesis of a waxy cuticle preceded the increase of several separation marker genes, *CELs* or *PGs,* in all three abscission systems. It was of particular interest that expression of soybean *GDSL*-like *lipase* genes that are known to be important for cuticle synthesis [78] and also *CER4* (Jojoba acyl CoA reductase) [79] and *LTPs* were greatly increased in an AZ-specific manner. Similar changes were seen in tomato and Arabidopsis where there was an AZ-specific up-regulation of gene expression for the tomato homologs of *CER4* (Jojoba acyl CoA reductase) and *LTPs*, and Arabidopsis homologs for *GDSL*-like *lipase*, and *bifunctional inhibitor*/*lipid*-*transfer protein* [43]. Thus, the data collectively suggest that the physical protection against pathogens and water loss [80] mediated through cuticle-like formation are crucial to organ separation in diverse systems [81]. It is worthy to note that formation of a cuticle-like substance plays a role in the separation of organs in meristems suggesting a similar role as observed in abscission [75,76,77,82].

These data indicate that the metabolism and regulatory modules utilized in organ separation in meristems are conserved in the separation of organs in a diverse set of abscission systems. This is functionally attributed to Phase 3 and Phase 4, which are the final phases of abscission as denoted in the model for abscission (Figure 1) [12,43,75,76,77,82]. How multiple signals including hormones, a small secreted peptide (e.g., inflorescence deficient in abscission, IDA), and environmental cues (i.e., light, heat, drought, frost, wind) interact to regulate the synthesis of extracellular components including the waxy cuticle to form a flexible extracellular layer in the AZ remains to be determined [37].

## 4. Transcriptional Regulatory Networks in the Soybean AZ

Biochemical changes required for clean separation obviously call for the hydrolysis of pectin and cellulose that unglue and loosen the middle lamella and primary cell wall, respectively [1,12,57]. In addition, a prerequisite for abscission to occur in response to abscission inducing signals like ethylene is a sensitization of the AZ cells resulting from a decline in auxin within the AZ [3,6,60,70]. As stated above, the working model for abscission consists of largely four basic phases [2,4,5,7,10,12,23,30,35,36,37]: first, differentiation of abscission zone (AZ); second, acquisition of the competence of the AZ to respond to abscission signals (e.g., decline in auxin); third, cell wall modification and cell separation; and fourth, trans-differentiation of the AZ and formation of a protective layer (Figure 1). Although this delineated model is instrumental to explain the culmination of organ separation in diverse abscission systems, as more data become available through high-throughput analyses, we are now beginning to understand more detailed biological processes that corroborate previous physiological and cytological observations [1,57].

Functional inferences using omics data (i.e., transcriptomic, proteomic, and metabolomic data) from many other plants have been generally adapted to predict gene function via guilt-by-association with Arabidopsis. Inferring gene functions, however, are often leveraged by many factors including the types of organs and plants studied, methods of sample collection, and analytic approaches, which all impact our interpretation and understanding of the biological processes utilized in abscission [43]. Further, there is a limited amount of information underlying interactions of multiple regulatory layers (e.g., transcriptional, post-transcriptional, and epigenetic regulation) that are required for a complex regulatory process necessary for abscission to occur [4,83]. Nevertheless, owing to considerable advancements in recent omics and subsequent bioinformatics, many abscission researchers have inevitably employed the omics-driven and informatics-supported inference tools to make considerable gains in an understanding of the cellular changes and molecular processes used in a diverse set of abscission systems [6,38,39,40,42,43,44,45]. All these studies highlight both conserved regulatory mechanisms as well as divergent mechanisms possibly due to different subsets of co-regulators required for successful organ separation depending on the organ and species studied [12,43,44].

### 4.1. Transcription Factors (TFs) in the AZ

**Figure 2:**
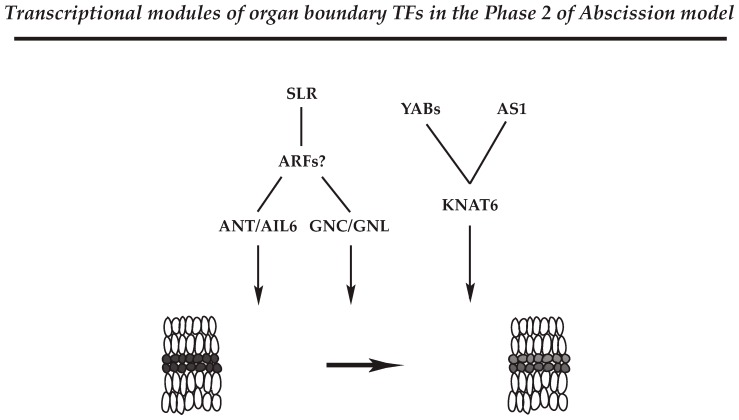
Representatives of the transcriptional modules associated with the formation of separation layer in the AZ of soybean leaf abscission. Transcription factors (TFs) that may define the separation layer in the AZs (Phase 2 possibly through Phase 3) are shown with their cognate regulators. Although *Auxin response factors 2* and *7* (*ARF2*/*7*) were not identified in the transcriptome data of soybean leaf abscission, gene expression of its upstream regulator, *SOLITARY ROOT* (*SLR*), was up-regulated at the onset of abscission, Phase 2 (Table 1 in Kim et al., 2016). The representative modules constitute ANT/AIL6 and GNC/GNL TFs that are possibly regulated by ARFs and their upstream regulator of SLR, which balance between cell proliferation and differentiation in the AZ at the onset of abscission. In addition, YAB and AS1 TFs may control the expression of *KNAT6* gene to regulate Phase 2 of abscission through the establishment of separation layer cells within the AZ.

The regulation of gene expression by transcription factors is a key mechanism that controls cellular changes in the AZ in response to developmental and environmental cues. Gene expression analysis for transcription factors (TFs) and their interacting proteins identified in the AZs has revealed regulatory modules embedded in the abscission network. For instance, functional inferences made for the soybean transcriptional landscape that govern leaf abscission has been reported [44]. In that study, homologs for many transcription factors (e.g., MYB, Zinc finger, bHLH, AP2, NAC, WRKY, YABBY, IAA), which were originally identified in the Arabidopsis genome, were represented as abscission-specific transcriptional regulators in soybean leaf abscission. In an attempt to dissect and highlight the connectivity of the biological processes of abscission, the authors clustered the expression profiles for TFs expressed in two consecutive time collections (i.e., 0 h and 12 h, 12 h and 24 h, 24 h and 48 h, and 48 h and 72 h) and then compared the expression in the leaf AZ (LAZ) to the non-abscission adjacent petiole tissues (NAZ) (i.e., LAZ/NAZ, AZ-specificity). The study revealed seven AZ-specific clusters that represent TF gene expression in the delineated phases of soybean leaf abscission from Phase 2 to Phase 4 (Figure 1). Of the seven clusters identified, the largest TF cluster was the first cluster that was up-regulated early in the abscission process, which contained YABBY (YAB) members (INNER NO OUTER (INO), ABNORMAL FLORAL ORGAN (AFO)/FILAMENTOUS FLOWER (FIL), YAB2, and YAB5). Functional studies of Arabidopsis *YAB* genes suggested that the *YAB* genes are critical in establishing organ polarity and cell identity by negative regulation of shoot apical meristem (SAM) genes that consequentially affect the growth of leaves, sepals, petals, and carpels [84,85,86,87,88,89,90]. The current annotation for the YAB TF gene family identifies 6 members with 8 gene models in Arabidopsis [84] (Plant TF at http://plntfdb.bioetanol.cnpem.br/v3.0/) and 17 members with 34 gene models in soybean (PlantTFDB at http://planttfdb.cbi.pku.edu.cn). Overrepresentation of YAB TFs in the differentially expressed genes of soybean abscission suggests that these TFs may be linked to organ polarity and identity of separation cells (i.e., the target cells) in the soybean leaf AZs (Figure 2) [44,84,85,87,88,89,90]. Notably, more than one third of the YAB TFs (6 out of 17) are strongly expressed in TF Cluster 1.

The same TF expression profile (Cluster 1) included homologs of HOMEOBOX 1 (ATHB-1) that are known to regulate cell fate and LATE MERISTEM-IDENTITY-1 (LMI1/HOMEOBOX 51/ATHB-51) that regulates organ identity in the meristem. A previous study demonstrated that the Arabidopsis homolog Homeobox 1 (ATHB-1) restricts the growth between floral organs and flower receptacle [91]. Although it remains to be experimentally determined, based on expression profiles, it appears that the TFs identified in this largest cluster of mostly up-regulated AZ-specific expression are functionally related to Phase 2 of abscission, which is the initial response of target cells to an abscission signal (Figure 1 and Figure 2) [44].

### 4.2. Regulatory Modules in Abscission

The omics-scale profiling of downstream transcriptional changes in abscission are being extensively studied; however, information on these regulatory networks that link TFs with cognate co-regulators is far from complete for a large spectrum of plant species other than Arabidopsis [92]. Nevertheless, systematic analyses in various species demonstrated that there are common attributes of TF regulatory networks having similar structures [93]. Importantly, these transcriptional regulatory networks are shown to constitute key regulatory hubs that control diverse biological processes [94,95,96,97,98]. Transcriptional regulatory networks of the soybean leaf AZ provide an additional example of common attributes that are associated with differing phases of abscission [44].

Extrapolating from the data in the Arabidopsis Transcriptional Regulatory Map (ATRM) [98], the largest regulatory network discovered in the soybean leaf abscission results revealed connectivity between TFs and co-regulators in a major regulatory hub that includes AINTEGUMENTA (ANT), AINTEGUMENTA-like 6 (AIL6), homeobox genes (e.g., KNOTTED-like 6 (KNAT6), ABARRENT TESTA SHAPE/KANADI 4 (ATS/KAN4), homeobox 51, BEL1), YAB (e.g., INO, AFO, YAB5), zinc finger (e.g., GATA, NITRATE-INDUCIBLE, CARBON-METABOLISM INVOVLED (GNC), CYTOKININ-INDUCED GATA1/GNC-like (GNL)), and Trihelix (e.g., PETAL LOSS (PTL) [44]. This largest module of TFs and their interacting proteins can be broadly interpreted as follows in the subsections below.

#### 4.2.1. Regulatory Module for Cell Proliferation and Differentiation

ANT and AIL TFs belong to a larger family of AP2/ERF TF family [99,100]. ANT and AIL genes control the balance between cell proliferation and differentiation in response to auxin gradients that define growth and patterning in different developmental processes [101]. In addition, ANT and AIL6 are associated with the maintenance of shoot and flower meristems, organ size, flower initiation, and floral organ identity. In the soybean abscission system, roles of ANT and AIL6 appear complex; however, as ANT and AIL6 are regulated by the AUXIN RESPONSE FACTOR 2 (ARF2) in Arabidopsis, it would appear that the ANT/AIL module may be associated with balancing between cell proliferation and differentiation in the soybean leaf AZ through translating the decline in auxin that occurs at the onset of abscission (Figure 2) [12,44,102].

#### 4.2.2. Regulatory Module for Integration of Other Hormone Signaling

Gene expression of GNC/GNL are induced by exogenous nitrate, cytokinin, and light treatments [103,104], and previous results demonstrated that GNC and GNL are negative regulators of various aspects of plant growth and development, including germination, GA catabolism, flowering time, senescence, and floral organ abscission in Arabidopsis [105,106]. In addition, it was reported that constitutive expression of GNC and GNL resembles the Arabidopsis *arf2* mutant phenotype that had defects in floral organ abscission [106,107,108]. Curiously, the transcriptome data for soybean leaf abscission identified *ARF8* but did not identify *ARF2*/*7* in the largest Cluster 1, which represents the onset of abscission. However, soybean homologs for Arabidopsis SOLITARY ROOT (SLR) that are upstream regulators of ARF7 in floral organ abscission [105,106] were strongly up-regulated in the Cluster 1. Thus, the commonality of phenotypes observed in Arabidopsis emphasizes the likelihood of a similar and significant role for GNC and GNL in the abscission process of soybean and other species. The results support a model where the plant hormones auxin and GA modulate GNC and GNL expression through transcriptional activities of ARF2 and ARF7. Further, ANT and AIL6, like GNC and GNL, are shown to be downstream components of the ARF2/7-mediated signaling module. Based on genetic and gene expression studies from Arabidopsis and soybean, the auxin transcription factors ARF2/ARF7 appear to mediate the co-transcriptional regulators of GNC/GNL and ANT/AIL6 that ultimately control abscission in plants (Figure 2). The results suggest that physiological programs associated with plant hormones integrate environmental signals to control plant growth and development including organ separation.

#### 4.2.3. Regulatory Module for Organ Polarity and Separation Boundary Determination

Analysis of the transcriptional networks in soybean leaf abscission further revealed that the above regulatory circuits that include ANT/AIL6 and GNC/GNL are also associated with organ polarity and boundary determinants [44]. Activities of YAB and KAN are known to regulate development and growth of plant organs including leaves, sepals, petals, and carpels [85,86,87,109]. KAN belongs to a larger family of GARP (GOLDEN2, ARR-B Class, Par1 proteins) [110]. It has been demonstrated that the activity of ATS/KAN4 is fundamental to the establishment of organ polarity and creation of organ boundaries [111]. As gene expression of soybean ATS/KAN4 homologs is increased during the actual cell separation (at 24 h and 48 h), it would be interesting to know if these genes are also involved in defining the separation boundary in the AZ.

The YAB family (AFO/FIL, YAB3, YAB2, YAB5) represses the expression of shoot apical meristem (SAM) regulatory genes [112], and de-repression of SAM regulatory genes results in SAM-like structures in Arabidopsis leaves [90,113]. In soybean leaf abscission, expression of the YAB TFs (INO, AFO/FIL, YAB2, YAB5) was strongly up-regulated at the beginning of abscission (at 0 h and 12 h) in an AZ-specific fashion (LAZ/NAZ). In addition, a slight up-regulation in expression for a soybean *KNAT6* was associated with the beginning of abscission at 12 h, and yet another *KNAT6* gene was slightly down-regulated later in abscission at 24 h. How exactly the regulatory module of YAB-KNAT6 functions in the AZ remains unclear; nonetheless, an AZ-specific expression of these genes early in the abscission suggests that YAB-KNAT6 may be associated with defining the separation boundary by suppressing AZ cell proliferation or promoting differentiation of AZ cells from Phase 2 through Phase 3 of the abscission model (Figure 2).

In Arabidopsis, ASYMMETRIC 1 (AS1) has multiple functions associated with organ boundary, polarity, cell fate, and the establishment of floral organ AZ [114,115,116]. In the Arabidopsis AZ, it appears that activity of AS1 is linked to the proper organization and timing of floral organ development (e.g., sepal, petal) [116]. Similar to the role of YABs, AS1 and its relative AS2 repress expression of *KNOTTED1-LIKE HOMEODOMAIN* (*KNOX*) genes, which regulate gene expression in the meristem [117,118,119]. In the soybean abscission system, expression of the soybean *AS1* homolog is up-regulated between 12 and 24 h at the beginning of cell separation and expression of soybean *KANT6* was down-regulated early in abscission; thus, it would be of experimental interest to know if soybean AS1 also controls the expression of the *KNAT6* genes to regulate abscission through cell differentiation and/or establishment of boundaries within the AZ (Figure 2).

## 5. Concluding Remarks and Future Perspectives

The biosynthesis and modification of plant cell walls play crucial roles throughout the lifecycle of plants. Mining of recent transcriptomic data has led to the discovery of novel aspects as to cell wall modifications that include the biosynthesis of a new extracellular matrix and transcriptional regulatory networks in the plant abscission system. Analysis of the transcriptomic data provides information to interpret the functional relevance of their expression and to better understand the complex molecular processes used in developmental processes and response to stress, including abscission. High throughput sequencing and expression profiling of AZ tissue of the soybean leaf abscission system in conjunction with informatics analyses have enabled inferences of regulatory networks that may be common to plant organ separation. In particular, identification of meristem-associated genes functionally associated with organ polarity, cell proliferation and differentiation, and cell identity in the AZs is of special interest for experimental validation of their role in abscission. Confirmation of the role of these basic molecular mechanisms in abscission will provide information for application of this knowledge to improve fruit quality and productivity in agriculturally important crops.
